# Association Between Charlson Comorbidity Index Items and Outcomes in Patients With Blunt Trauma Using a Nationwide Trauma Registry Database in Japan

**DOI:** 10.7759/cureus.95974

**Published:** 2025-11-02

**Authors:** Sayaka Noguchi, Daizoh Saitoh, Hideharu Tanaka

**Affiliations:** 1 Graduate School of Emergency Medical System, Kokushikan University, Tokyo, JPN; 2 Department of Emergency Medicine, Kyoto Tachibana University, Kyoto, JPN; 3 Department of Traumatology and Critical Care Medicine, National Defense Medical College, Tokorozawa, JPN

**Keywords:** blunt trauma, charlson comorbidity index, cox regression analysis, in-hospital outcome, japan trauma data bank

## Abstract

Objective: Understanding a patient's medical history and comorbidities before arrival at a hospital can help select the appropriate medical institution for transport and may be related to outcomes by assisting with treatment after arrival at the hospital. This study aimed to clarify the comorbidities associated with blunt trauma outcomes.

Methods: Using the Japan Trauma Data Bank (JTDB), a nationwide large dataset in Japan, we studied 119,929 cases of blunt trauma among patients admitted to JTDB-participating hospitals from January 2019 to December 2022, excluding those with cardiac arrest before hospital arrival. We compared the groups according to the severity of the trauma, age, sex, and each item of the original Charlson Comorbidity Index (CCI) at the time of discharge and then used Cox regression analysis, a multivariate analysis, to explore the relationship between the outcome and each item of the CCI.

Results: There were significant differences in 10 of the 19 CCI items between the mortality and survival groups. However, Cox regression analysis, including the bootstrap method, was narrowed down to seven items, and the comorbidities that were significantly associated with mortality were leukemia, moderate or severe liver disease, metastatic malignant neoplasm, moderate renal disease, congestive heart failure, collagen disease, and chronic pulmonary disease in that order.

Conclusion: Comorbidities that can affect the prognosis of blunt trauma were identified from the items in the original CCI. It is beneficial for emergency medical personnel to know about pre-existing comorbidities associated with the prognosis of blunt trauma cases when selecting a transport facility before arrival at the hospital, and the condition of trauma cases with pre-existing or dysfunctional major organs, such as the liver, kidneys, heart, and lungs, should not be underestimated.

## Introduction

Background

Gathering information about a patient's medical history and comorbidities is necessary when collecting prehospital information [1-4]. This information may be relevant to the cause of the patient's symptoms [1], and knowing the hospital that they usually visit may be the basis for choosing a hospital [2]. In addition, providing doctors with information about the patient's medical history and comorbidities when they are transported to the hospital may help speed up treatment after they arrive [3]. Furthermore, in cases of trauma, it is necessary to consider that comorbidities may affect the outcome of the patient's condition [4]. One way to do this would be to utilize big data, such as the Japan Trauma Data Bank (JTDB) [5,6], to identify pre-existing conditions and comorbidities that affect the outcome of blunt trauma so that emergency medical technicians can ask about pre-existing conditions and comorbidities at the scene and select a more appropriate facility for transport, which can potentially improve patient survival rates.

The Charlson Comorbidity Index (CCI) [7] is a relatively simple index designed to weigh the severity of a patient's comorbidities and predict future mortality risk. It was developed by Mary Charlson in 1987 and covers 19 diseases. This index is widely used in clinical research and has long played an essential role in research design. However, this is not a trauma-specific comorbidity index [8,9]. The original CCI, created in 1987, covers a wide range of diseases, and there are revised indices for specific diseases [10].

Importance

The JTDB contains information on the 19 items of the original CCI, a standard comorbidity index that is not specific to trauma [7]. Although there are research reports on the relationship between the total CCI score and outcomes of trauma cases [11], a study conducting a detailed analysis using each CCI item has not yet been conducted. In addition, gunshot wounds are extremely rare in Japan [12], and the situation regarding blunt trauma and the quality of trauma differs from that in other countries where gunshot wounds are relatively common. This study focused on blunt trauma, which is also applicable to international research. However, the extent to which diseases in the CCI items are associated with the outcomes of blunt trauma has not been clarified.

Goals of this investigation

This study aimed to analyze the comorbidities that affect the in-hospital mortality of blunt trauma using the 19 items of the original CCI in the JTDB and to obtain helpful comorbidity information for paramedics when selecting a hospital to transport patients.

## Materials and methods

Research design

This study was a retrospective cohort study using the JTDB database. The JTDB [5,6] is a nationwide trauma registry established by the Japanese Society of Trauma Medicine and the Japanese Association of Emergency Medicine to improve the quality of trauma care in Japan. The number of participating hospitals has been increasing annually; as of April 2023, 303 hospitals have joined the JTDB data registry. In addition, the JTDB migrated from the old system to a new one in 2019. This study was published in 2023 and used case data from the new system for admissions to JTDB-participating registered facilities from 2019 to 2022.

Ethics declarations

This study was conducted in accordance with the principles of the Declaration of Helsinki. The Kokushikan University Ethics Committee approved the retrospective cohort study using JTDB data (approval number: 24009).

Study endpoints

The primary endpoint was the in-hospital mortality of blunt trauma cases. The secondary outcome was the identification of CCI components significantly associated with in-hospital mortality in blunt trauma cases.

Patient selection

From the new JTDB system, 133,384 patients were admitted from January 1, 2019, to December 31, 2022. We excluded other instances than blunt trauma cases and excluded cases with cardiopulmonary arrest at the time of emergency medical services (EMS) contact at the scene and cases with cardiopulmonary arrest on arrival at the hospital; blunt trauma cases were included in this study. In addition, cases were compared and analyzed by dividing them into survivors and fatalities as outcomes at hospital discharge.

Data used

Among the available items in the data studied in the new JTDB system, the following variables were used: age, sex, type of trauma, route of transport, method of transport, systolic blood pressure at hospital arrival, Glasgow Coma Scale [13] score at prehospital and hospital arrival, Revised Trauma Score (RTS) [14], Injury Severity Score (ISS) [15], Abbreviated Injury Scale (AIS) [16] score, AIS 2005 update 2008 (hereafter AIS 2008) maximum score for body part 9, total number of CCI scores indicating history and comorbidities [7] and each component (based on the definition of comorbidities of original CCI methodology) [7], days of hospitalization, and discharge outcomes (survival and death).

Statistical analyses

Blunt trauma cases were presented by age, sex, route of transport, method of transport, CCI score, RTS, ISS, and case occupancy for each of the nine AIS domains, with a maximum score of ≥3 based on discharge outcome. In addition, a two-arm comparison was performed for the case occupancy of each CCI domain in the survivor and death groups.

Survival analysis of the study cases was performed using Cox regression analysis. The criterion variable was the number of survival days (0 d if the patient died on the day of the injury). The explanatory variables were age; sex (male: 0; female: 1); ISS, RTS, and AIS regions 1-9, with a maximum score of 0-2 categorized as 0 and 3-6 as 1; and the presence of each CCI item. The results are presented as standardized coefficients, hazard ratios, p-values, and 95% confidence intervals (CIs). Additionally, we used the bootstrap method to evaluate the statistical stability and robustness of the CIs. In other words, we performed 1,000 iterations of random sampling and Cox regression analysis on each bootstrap sample to obtain the distribution of the regression coefficients and to calculate the 95% CI.

Categorical variables are presented as numbers and percentages, and quantitative variables are presented as means or medians (first-third quartile, interquartile range (IQR)). The chi-squared test was used to compare categorical variables, and the Mann-Whitney U test was used to compare quantitative variables. Statistical significance was defined as a two-sided p-value of <0.05 for all statistical analyses. JMP Pro 15.0.0 (SAS Institute, Cary, North Carolina, United States) and IBM SPSS Statistics for Windows, Version 29.0 (IBM Corp., Armonk, New York, United States), were used for statistical analyses.

## Results

**Figure 1:**
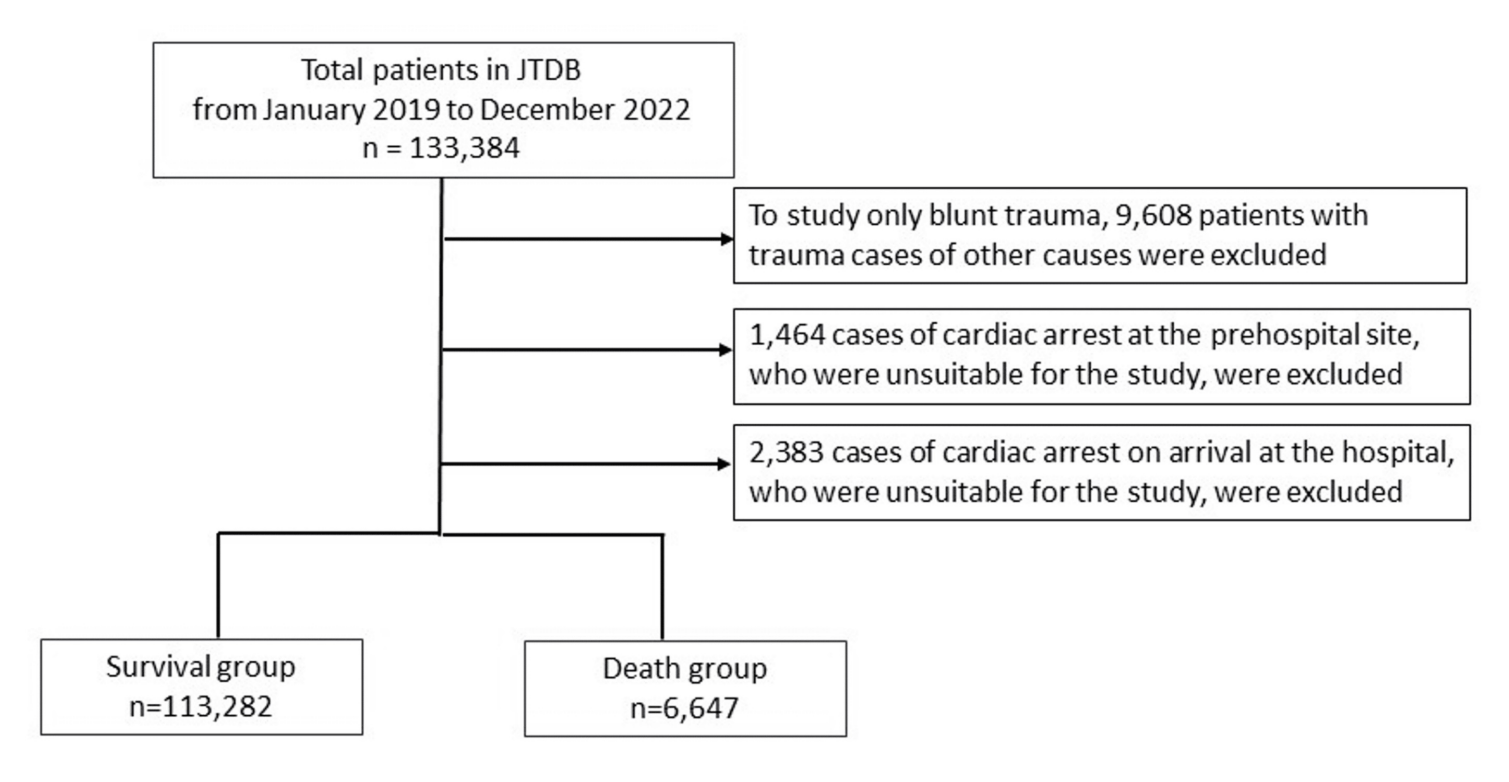
Study flow diagram of the patients included JTDB: Japan Trauma Data Bank; CCI: Charlson Comorbidity Index; EMS: emergency medical services; RTS: Revised Trauma Score; ISS: Injury Severity Score; AIS: Abbreviated Injury Scale; 95% CIs: 95% confidence intervals; IQR: interquartile range; SRC: standardized regression coefficient; HR: hazard ratio

Figure 1 presents a flowchart of the 133,384 cases selected for this study. Of the 133,384 cases registered in the new JTDB database released in 2023, 9,608 cases other than blunt trauma, which were penetrating trauma, burns, combined injuries, and cases with unknown mechanism of injury, were excluded. In addition, 119,929 blunt trauma cases were included in this study, excluding 1,464 cases of cardiopulmonary arrest at the EMS scene contact and 2,383 cases of cardiopulmonary arrest at hospital arrival. The outcomes at discharge included survival in 113,282 patients (94.5%) and death in 6,647 patients (5.5%).

Patient backgrounds in the death and survival groups, grouped based on outcomes at discharge, are shown in Tables 1-3. The male sex was predominant, with 4,371 (65.8%) patients in the death group and 64,274 (56.7%) in the survival group. On the other hand, the female sex was 2,200 (33.1%) patients in the death group and 47,842 (42.2%) in the survival group in Table 1. The mean ages were 71.6 years in the survival group and 63.9 years in the control group in Table 2. Regarding the route of transport to hospital arrival, more deaths were transported directly from the scene than survivors, and 95,242 cases (79.4%) were brought in by ambulance as the method of transport in Table 1. The CCI total score, ISS, and RTS are shown in Table 2. However, the median (IQR) values are also shown because some items were difficult to distinguish between the two groups. In Table 2, the mean values for the deceased and surviving groups were CCI total scores of 0.83 and 0.61, ISS of 24.6 and 12.4, and RTS of 5.67 and 12.4, respectively. The CCI total score and ISS were higher in the death group than in the survival group and were lower in the RTS group; therefore, the death group had higher trauma severity than the survival group. The number and percentage of cases with an AIS of ≥3 in each of the nine regions are also presented in Table 3. In domains 1 (head), 2 (face), 3 (neck), 5 (abdomen), and 9 (body surface/others), the death group tended to have a higher case share of AIS maxima ≥3 than the survival group, whereas in domains 4 (chest), 6 (spine), 7 (upper extremity), and 8 (lower extremity, pelvis, and hip), the death group tended to have a lower case occupancy rate of AIS maxima ≥3 than the surviving group.

**Table 1:** Profile of cases with blunt trauma, classified by backgrounds and outcome Total: 119,929 cases. Death group: 6,647 cases. Survival group: 113,282 cases JTDB: Japan Trauma Data Bank; CCI: Charlson Comorbidity Index; EMS: emergency medical services; RTS: Revised Trauma Score; ISS: Injury Severity Score; AIS: Abbreviated Injury Scale; 95% CIs: 95% confidence intervals; IQR: interquartile range; SRC: standardized regression coefficient; HR: hazard ratio

Variable	Total (n)	{%}	Death (n)	{%}	Survival (n)	{%}
Gender						
Male	68,645	{57.2}	4,371	{65.8}	64,274	{56.7}
Female	50,042	{41.7}	2,200	{33.1}	47,842	{42.2}
Transport route						
From scene	91,697	{76.5}	5,537	{83.3}	86,160	{76.1}
From hospital	19,439	{16.2}	746	{11.2}	18,693	{16.5}
From others	5,512	{4.6}	169	{2.5}	5,343	{4.7}
Unknown	3,281	{2.7}	195	{2.9}	3,086	{2.8}
Transport means						
Ambulance	95,242	{79.4}	5,357	{80.6}	89,885	{79.3}
Doctor car	4,228	{3.5}	395	{5.9}	3,833	{3.4}
Doctor helicopter	5,970	{5.0}	490	{7.4}	5,480	{4.8}
Hospital car	925	{0.8}	26	{0.4}	899	{0.8}
Private car	4,054	{3.4}	41	{0.6}	4,013	{3.5}
Other/unknown	9,510	{7.9}	338	{5.1}	9,172	{8.1}

**Table 2:** Profile of cases with blunt trauma, classified by trauma severity and outcome Total: 119,929 cases. Death group: 6,647 cases. Survival group: 113,282 cases JTDB: Japan Trauma Data Bank; CCI: Charlson Comorbidity Index; EMS: emergency medical services; RTS: Revised Trauma Score; ISS: Injury Severity Score; AIS: Abbreviated Injury Scale; 95% CIs: 95% confidence intervals; IQR: interquartile range; SRC: standardized regression coefficient; HR: hazard ratio

Variable	Total mean	Median	IQR	Death mean	Median	IQR	Survival mean	Median	IQR
Age: mean, median (IQR)	64.4	71	(50-83)	71.6	78	(64-86)	63.9	71	(49-83)
CCI	0.63	0	(0-1)	0.83	0	(0-1)	0.61	0	(0-1)
RTS	7.5	7.84	(7.84-7.84)	5.67	5.97	(4.09-7.84)	12.4	7.84	(7.84-7.84)
ISS	13.1	9	(9-17)	24.6	25	(16-29)	12.4	9	(9-16)

**Table 3:** Profile of cases with blunt trauma, classified by AIS max score and outcome Total: 119,929 cases. Death group: 6,647 cases. Survival group: 113,282 cases JTDB: Japan Trauma Data Bank; CCI: Charlson Comorbidity Index; EMS: emergency medical services; RTS: Revised Trauma Score; ISS: Injury Severity Score; AIS: Abbreviated Injury Scale; 95% CIs: 95% confidence intervals; IQR: interquartile range; SRC: standardized regression coefficient; HR: hazard ratio

Variable	Total (n)	{%}	Death (n)	{%}	Survival (n)	{%}
AIS						
Region 1 max score ≧3	26,523	{22.1}	4,091	{61.5}	22,432	{19.8}
Region 2 max score ≧3	497	{0.4}	79	{1.2}	418	{0.4}
Region 3 max score ≧3	228	{0.2]	19	{0.3}	209	{0.2}
Region 4 max score ≧3	22,934	{19.1}	930	{14.0}	21,004	{18.5}
Region 5 max score ≧3	4,588	{3.8}	471	{7.1}	4,117	{3.6}
Region 6 max score ≧3	13,396	{11.2}	612	{9.2}	12,784	{11.3}
Region 7 max score ≧3	1,268	{1.1}	38	{0.6}	1,230	{1.1}
Region 8 max score ≧3	40,180	{33.5}	1,510	{22.7}	38,670	{34.1}
Region 9 max score ≧3	517	{0.4}	148	{2.2}	369	{0.3}
Unclear cases	3880	{3.2}	201	{3.0}	3,679	{3.2}

Table 4 shows the results of the comparison between the two groups for each CCI item. In other words, it shows the rate of pre-existing complications and comorbidities for each CCI item in the death and survival groups. Items showing significant differences between the two groups, in order of death group and survival group, were as follows: myocardial infarction: 226 cases (3.40%) and 2,471 cases (2.18%); congestive heart failure: 295 cases (4.44%) and 2,878 cases (2.54%); cerebrovascular disease: 477 cases (7.18%) and 6,803 cases (6.01%); mild liver disease: 111 cases (1.67%) and 1,542 cases (1.36%); diabetes with end-organ damage: 104 cases (1.57%) and 1,361 cases (1.20%); moderate or severe renal impairment: 268 cases (4.03%) and 2,431 cases (2.15%); any tumor: 334 cases (5.03%) and 4,549 cases (4.02%); leukemia: 38 cases (0.57%) and 165 cases (0.15%); moderate or severe liver disease: 51 cases (0.77%) and 244 cases (0.22%); and metastatic solid tumor: 120 cases (1.81%) and 704 cases (0.62%). Although the results were not adjusted for illness severity in each case, 10 of the 19 items showed significant differences at p<0.05.

**Table 4:** Comparisons of CCI items between the two groups Total: 119,929 cases. Death group: 6,647 cases (5.5%). Survival group: 113,282 cases (94.5%) JTDB: Japan Trauma Data Bank; CCI: Charlson Comorbidity Index; EMS: emergency medical services; RTS: Revised Trauma Score; ISS: Injury Severity Score; AIS: Abbreviated Injury Scale; 95% CIs: 95% confidence intervals; IQR: interquartile range; SRC: standardized regression coefficient; HR: hazard ratio

Variable	Missing (n)	Total (n)	{%}	Death (n)	{%}	Survival (n)	{%}	P-value
Myocardial infarct	171	2,697	{2.25}	226	{3.40}	2,471	{2.18}	<0.0001
Congestive heart failure	172	3,173	{2.65}	295	{4.44}	2,878	{2.54}	<0.0001
Peripheral vascular disease	176	828	{0.69}	58	{0.87}	770	{0.68}	0.0658
Cerebrovascular disease	171	7,280	{6.08}	477	{7.18}	6,803	{6.01}	<0.0001
Dementia	174	10,714	{8.95}	625	{9.40}	10,089	{8.92}	0.1735
Chronic pulmonary disease	174	3,448	{2.88}	203	{3.06}	3,245	{2.87}	0.3745
Connective tissue disease	174	1,195	{1.00}	61	{0.92}	1,134	{1.00}	0.5025
Ulcer disease	173	1,727	{1.44}	98	{1.48}	1,629	{1.44}	0.8145
Mild liver disease	173	1,653	{1.38}	111	{1.67}	1,542	{1.36}	0.0366
Diabetes	165	13,735	{11.47}	789	{11.88}	12,946	{11.44}	0.282
Diabetes with end-organ damage	174	1,465	{1.22}	104	{1.57}	1,361	{1.20}	0.009
Moderate or severe renal damage	176	2,699	{2.25}	268	{4.03}	2,431	{2.15}	<0.0001
Hemiplegia	175	1,479	{1.24}	77	{1.16}	1,402	{1.24}	0.5652
Any tumor	174	4,883	{4.08}	334	{5.03}	4,549	{4.02}	<0.0001
Leukemia	175	203	{0.17}	38	{0.57}	165	{0.15}	<0.0001
Lymphoma	175	457	{0.38}	32	{0.48}	425	{0.38}	0.1731
Moderate or severe liver disease	175	295	{0.25}	51	{0.77}	244	{0.22}	<0.0001
Metastatic solid tumor	175	824	{0.69}	120	{1.81}	704	{0.62}	<0.0001
AIDS	175	49	{0.04}	4	{0.06}	45	{0.04}	0.4232

The results of the Cox regression analysis, with survival days as the objective variable, are presented in Table 5. The Cox proportional hazards model was statistically significant (χ²=19545.470; df=32; p<0.001), indicating improved model fit over the null model (-2 log likelihood=76186.726). The order of the absolute values of the standard regression coefficients and magnitude of the hazard ratios for each CCI item was as follows: leukemia, moderate or severe liver disease, metastatic malignant neoplasms, moderate renal disease, congestive heart failure, collagen disease, chronic lung disease, and mild liver disease. Useful history and comorbidities were identified in eight of the 19 items in the CCI. Excluding malignant diseases, leukemia, and metastatic malignant neoplasms, patients with comorbidities of the liver, kidney, heart, and lungs had significantly poor outcomes.

**Table 5:** Results of the Cox regression analysis JTDB: Japan Trauma Data Bank; CCI: Charlson Comorbidity Index; EMS: emergency medical services; RTS: Revised Trauma Score; ISS: Injury Severity Score; AIS: Abbreviated Injury Scale; 95% CIs: 95% confidence intervals; IQR: interquartile range; SRC: standardized regression coefficient; HR: hazard ratio

Variable	SRC	p	HR	CI
Age	0.03	<0.001	1.031	1.029-1.033
Gender (male)	-0.245	<0.001	0.783	0.730-0.840
RTS	-0.642	<0.001	0.526	0.516-0.537
ISS	0.029	<0.001	1.03	1.027-1.033
AIS body region 1 max score ≧3	0.77	<0.001	2.159	1.977-2.357
AIS body region 2 max score ≧3	-0.015	0.919	0.985	0.734-1.322
AIS body region 3 max score ≧3	-0.114	0.695	0.892	0.504-1.578
AIS body region 4 max score ≧3	-0.239	<0.001	0.788	0.726-0.855
AIS body region 5 max score ≧3	0.294	<0.001	1.342	1.181-1.526
AIS body region 6 max score ≧3	-0.722	<0.001	0.486	0.427-0.553
AIS body region 7 max score ≧3	-0.613	0.003	0.542	0.362-0.811
AIS body region 8 max score ≧3	-0.492	<0.001	0.612	0.561-0.667
AIS body region 9 max score ≧3	0.057	0.692	1.059	0.799-1.404
Myocardial infarct	0.039	0.656	1.04	0.876-1.234
Congestive heart failure	0.501	<0.001	1.65	1.421-1.916
Peripheral vascular disease	-0.249	0.214	0.78	0.527-1.154
Cerebrovascular disease	0.059	0.313	1.061	0.945-1.191
Dementia	0.093	0.091	1.097	0.985-1.221
Chronic pulmonary disease	0.314	<0.001	1.369	1.154-1.624
Connective tissue disease	0.318	0.031	1.374	1.030-1.833
Ulcer disease	-0.062	0.652	0.94	0.718-1.231
Mild liver disease	0.254	0.03	1.289	1.025-1.621
Diabetes	-0.043	0.375	0.958	0.872-1.053
Diabetes with end-organ damage	-0.131	0.305	0.878	0.684-1.127
Moderate or severe renal damage	0.622	<0.001	1.863	1.589-2.184
Hemiplegia	-0.177	0.223	0.837	0.630-1.114
Any tumor	0.001	0.989	1.001	0.867-1.155
Leukemia	1.268	<0.001	3.553	2.400-5.261
Lymphoma	0.184	0.399	1.202	0.784-1.845
Moderate or severe liver disease	1.137	<0.001	3.119	2.288-4.250
Metastatic solid tumor	1	<0.001	2.717	2.179-3.389
AIDS	-1.567	0.146	0.209	0.025-1.727

Table 6 presents the results of the bootstrap analysis. The adjusted hazard ratios and 95% CIs for all parameters (except mild liver disease) were identified as independent prognostic factors. In other words, in the 1,000 iterations of the bootstrap analysis, all items except mild liver disease that were significant in the original Cox regression analysis also retained statistical significance in the bootstrap analysis results, indicating the stability of the prognostic prediction ability of these factors. Regarding the item of mild liver disease, the hazard ratio was 1.289, maintaining its eighth place in the weighting order as a result of the bootstrap method, but the 95% CI was 0.976-1.654, and the significance for the outcome survival period, the objective variable, disappeared; therefore, it was not a stable and significant item.

**Table 6:** Results using the bootstrap method of Cox regression analysis JTDB: Japan Trauma Data Bank; CCI: Charlson Comorbidity Index; EMS: emergency medical services; RTS: Revised Trauma Score; ISS: Injury Severity Score; AIS: Abbreviated Injury Scale; 95% CIs: 95% confidence intervals; IQR: interquartile range; SRC: standardized regression coefficient; HR: hazard ratio

Variable	SRC	p	HR	CI
Age	0.03	<0.001	1.031	1.028-1.034
Gender (male)	-0.245	<0.001	0.783	0.721-0.848
RTS	-0.642	<0.001	0.526	0.513-0.538
ISS	0.029	<0.001	1.03	1.026-1.035
AIS body region 1 max score ≧3	0.77	<0.001	2.159	1.919-2.375
AIS body region 2 max score ≧3	-0.015	0.928	0.985	0.703-1.327
AIS body region 3 max score ≧3	-0.114	0.763	0.892	0.388-1.642
AIS body region 4 max score ≧3	-0.239	<0.001	0.788	0.715-0.868
AIS body region 5 max score ≧3	0.294	0.002	1.342	1.116-1.602
AIS body region 6 max score ≧3	-0.722	<0.001	0.486	0.405-0.569
AIS body region 7 max score ≧3	-0.613	0.042	0.542	0.290-0.950
AIS body region 8 max score ≧3	-0.492	<0.001	0.612	0.547-0.675
AIS body region 9 max score ≧3	0.057	0.736	1.059	0.739-1.445
Myocardial infarct	0.039	0.701	1.04	0.836-1.283
Congestive heart failure	0.501	<0.001	1.65	1.399-1.944
Peripheral vascular disease	-0.249	0.242	0.78	0.483-1.157
Cerebrovascular disease	0.059	0.364	1.061	0.940-1.206
Dementia	0.093	0.105	1.097	0.979-1.226
Chronic pulmonary disease	0.314	0.005	1.369	1.088-1.667
Connective tissue disease	0.318	0.038	1.374	1.005-1.826
Ulcer disease	-0.062	0.681	0.94	0.701-1.230
Mild liver disease	0.254	0.058	1.289	0.976-1.654
Diabetes	-0.043	0.426	0.958	0.867-1.067
Diabetes with end-organ damage	-0.131	0.338	0.878	0.650-1.156
Moderate or severe renal damage	0.622	<0.001	1.863	1.523-2.300
Hemiplegia	-0.177	0.345	0.837	0.569-1.185
Any tumor	0.001	0.986	1.001	0.850-1.185
Leukemia	1.268	<0.001	3.553	2.519-5.254
Lymphoma	0.184	0.518	1.202	0.676-1.921
Moderate or severe liver disease	1.137	<0.001	3.119	2.065-4.914
Metastatic solid tumor	1	<0.001	2.717	2.090-3.466
AIDS	-1.567	0.264	0.209	0.0001-2.762

## Discussion

This study focuses on blunt trauma because, in Japan, sharp trauma is mainly caused by penetrating injuries from blades [17], whereas gunshot wounds are far more common in the United States than in Japan. To conduct trauma research that is of international significance, this study focused on blunt trauma and excluded sharp trauma, which is a situation unique to Japan, and performed an analysis. The aim was to obtain universally applicable results that were internationally valid by analyzing whether the individual comorbidities in each case were originally associated with the outcome.

The objective of this research was to extract medical history and comorbidities that significantly affected the outcomes of blunt trauma from the original CCI [7]. Only a limited number of academic papers have used the JTDB to report on the CCI [11], and we could not find any articles that reported on the outcomes of blunt trauma for each item of the original CCI within the scope of our search. In a simple comparison between the two groups by discharge outcome with no adjustment for covariates, 10 comorbidities showed a significant difference. However, in multivariate analysis using the Cox regression model for survival analysis, there were eight CCI items with a p-value of <0.05. In addition, as the bootstrap method did not produce significant results for mild liver disease (p=0.058), it is safe to say that there were seven items. These seven items are thought to be significantly associated with in-hospital mortality. Using the hazard ratios from the Cox regression analysis results in this study, it may be possible to create a new index of comorbidities specific to blunt trauma according to methods, such as those described by Quan et al. [10].

According to the absolute value of the standard regression coefficient or the magnitude of the hazard ratio, the weighting order associated with the death outcome, which was the target variable, was as follows: leukemia, moderate or severe liver disease, metastatic malignant neoplasm, moderate renal disease, congestive heart failure, collagen disease, and chronic pulmonary disease. Leukemia refers to acute or chronic myeloid leukemia, acute or chronic lymphocytic leukemia, or polycythemia vera. Moderate or severe liver disease refers to cirrhosis with portal hypertension. Metastatic malignancy refers to distant metastasis of solid tumors [7]. These are all severe comorbidities for trauma patients. Moderate renal disease refers to serum creatinine levels >3 mg/dL (including patients receiving hemodialysis) and congestive heart failure >2 in the New York Heart Association [7], so severe trauma patients may not survive the acute phase. Connective tissue disease refers to systemic lupus erythematosus, polymyositis, mixed connective tissue disease, polyangiitis, or moderate-to-severe rheumatoid arthritis [7]. The association with in-hospital mortality is unclear, but may be related to current or past use of oral corticosteroids. Chronic pulmonary disease, which is associated with moderate or severe activity and blood carbon dioxide retention or oxygen partial pressure <50 mmHg, causing respiratory distress, is understandable to be associated with in-hospital mortality in trauma patients [7].

Among the pre-existing comorbidities associated with these fatal outcomes, excluding malignant diseases, such as leukemia and metastatic malignant neoplasms, cases with organ damage to the liver, kidney, heart, or lungs had significantly poor outcomes. Of these organs, the heart, kidneys, and lungs are closely related to circulation. In addition, hemorrhagic shock is an essential factor in the cause of death from trauma [18]. From this perspective, having a pre-existing condition that may cause some circulatory disturbance before sustaining an injury could be one factor in a poor outcome. In addition, there are cases where severe liver damage can cause blood coagulation disorders [19], and this may contribute to hemorrhagic shock and may be associated with death in cases of blunt trauma.

Furthermore, when leukemia, moderate liver disease, and metastatic malignant neoplasms are the top three, they all have common underlying diseases that weaken resistance to bacterial infections. Even if patients with trauma overcome acute circulatory failure due to bleeding, they may develop bacterial infections from wounds in the subacute phase or later and die from sepsis or organ failure [20,21]. Therefore, pre-existing complications, such as leukemia, moderate liver disease, and metastatic malignant neoplasms, are thought to reduce immune function, weaken resistance to bacterial infections, and increase the risk of death.

The CCI is a standard comprehensive indicator of pre-existing conditions in medical literature [7], yet academic papers discussing its impact on outcomes in trauma cases are minimal [11]. Furthermore, within the scope of our review, we were unable to identify any papers specifically addressing the influence of each CCI pre-existing condition on patient outcomes. As Quan et al. modified the CCI to create a new version associated with in-hospital mortality [10], we believe a new comorbidity index is needed to aid prehospital triage for trauma patients. However, as we cannot entirely rule out the potential for age-related biases concerning comorbidities in this study's analysis, or the emphasis on prehospital comorbidities, to introduce potential harm in hospital selection triage, we shall continue our research into these aspects.

Limitations

First, as this was a retrospective study using the JTDB, there were missing values in some data items, and some observed values were not measured, depending on the case. Thus, when variables required for analysis were missing as data in the items for each case, that case was excluded from the analysis and processed accordingly. Second, as this study focused on blunt trauma, it did not provide useful information on sharp trauma, such as gunshot wounds, which account for a large proportion of trauma cases overseas. Third, Japan's prehospital emergency transport system has its own unique style, such as the emergency medical technician system [22]. Thus, unfortunately, it remains unclear whether it has universal significance from an international perspective. Fourth, differences in study target populations, Japanese healthcare systems, injury therapeutic measures across hospitals, and the influence of the COVID-19 pandemic may limit generalizability. Fifth, the prevalence of comorbidities is low, resulting in low detection power; further validation using other big data may be necessary.

## Conclusions

This study extracted seven comorbidities associated with the prognosis of blunt trauma cases from the original CCI items. It is beneficial for emergency medical personnel to know about pre-existing comorbidities associated with the prognosis of blunt trauma cases when selecting a transport facility before arrival at the hospital, and the condition of trauma cases with pre-existing or dysfunctional major organs, such as the liver, kidneys, heart, and lungs, should not be underestimated.
